# Quantification of smoking-related airway remodelling in COPD, using N-Tidal

**DOI:** 10.1038/s41598-026-41699-6

**Published:** 2026-02-26

**Authors:** Rui Hen Lim, Leeran Talker, Henry Broomfield, Cihan Dogan, Ahmed B. Selim, Gabriel Lambert, Julian C. Carter, Daniel M. Neville, Laura Wiffen, Thomas Brown, BRS Team, Jonathan Winter, Andrew Gribbin, Milan Chauhan, Ruth De Vos, Paul Kalra, Selina Begum, Elango Vijaykumar, Anoop J. Chauhan, Ameera X. Patel

**Affiliations:** 1 TidalSense Limited, Cambridge, UK; 2 Portsmouth Hospitals University NHS Trust, Portsmouth, UK; 3 Modality Partnership, Birmingham, UK

**Keywords:** Capnography, Chronic obstructive pulmonary disease, Smoking, Diseases, Health care, Medical research, Risk factors

## Abstract

Tobacco smoking is the primary cause of chronic obstructive pulmonary disease (COPD) globally. Capnography data was collected twice daily for up to 6 months from 147 COPD participants across multiple studies using TidalSense’s N-Tidal device. Waveform features from the alpha angle region of the capnogram showed strong association with pack year history, indicating that capnography can quantify a dose-response relationship between smoking exposure and airway remodelling. This non-linear association reached an inflection around 25 pack years, potentially indicating a ‘tipping point’ beyond which the likelihood of retaining normal lung function significantly diminishes. This provides valuable mechanistic insights and could help estimate disease risk and support early preventative interventions.

**Trial registration** ClinicalTrials.gov NCT02814253 (registered on 27 June 2016), ClinicalTrials.gov NCT03615365 (registered on 3 August 2018), ClinicalTrials.gov NCT04939558 (registered on 25 June 2021).

## Background

The global tobacco epidemic is a significant public health threat, causing over 8 million deaths annually and costing the global economy around USD 1.4 trillion each year in healthcare and lost productivity^1,2^. Over 80% of the 1.3 billion tobacco users live in low and middle-income countries, where the health and economic burden is particularly severe. Tobacco smoking is a leading cause of respiratory disease including chronic obstructive pulmonary disease (COPD) and lung cancer, and worsens conditions such as asthma, tuberculosis, and pneumonia^3^.

Capnography, which measures respired carbon dioxide (CO_2_), is an established clinical measure for assessing ventilation. However, its use in respiratory contexts has been limited by the availability of medical-grade high-resolution sensors. TidalSense’s N-Tidal handheld, portable device now enables reliable and accurate measurement of CO_2_ concentration with a high sampling frequency^4^. The objective of this research was to use capnography data collected by the N-Tidal device to assess whether cumulative smoking exposure could be quantified by the CO_2_ waveform itself in patients with a confirmed COPD diagnosis, potentially offering new insights into smoking-related airway remodelling.

## Methods

Capnography data in this study was collected from three longitudinal observational studies in the UK: CBRS (NCT02814253), CBRS2 (NCT03615365), and CARES (NCT04939558). These involved participants with COPD and other cardiorespiratory conditions; only participants with COPD were included in this analysis, all diagnosed according to the latest National Institute for Health and Care Excellence (NICE) guidelines^5^. All data collection was conducted in accordance with the principles of the Declaration of Helsinki. In all studies, participants used the N-Tidal Handset, a CE-marked medical device regulated in the EU that measures respired partial pressure of CO_2_ (pCO_2_) directly from the mouth during tidal breathing.

After training, participants recorded a CO_2_ breath record (one capnogram) during 75 s of normal tidal breathing, twice daily for between 2 weeks and 12 months. Medical histories, including smoking histories, were obtained. Individual study objectives, ethical approvals, participant recruitment, eligibility criteria and informed consent have been previously described, alongside the methods used for feature engineering^6^. Given that COVID-19-related sequelae can affect respiratory mechanics, study timings and inclusion criteria were reviewed to ensure accurate assessment of pulmonary function outcomes. The CBRS and CBRS2 studies were conducted before the pandemic, while the CARES study included a separate recruitment category for long COVID, allowing potential post-COVID effects to be identified.

Statistical analysis was conducted using Python (version 3.10.10). The relationships between the capnography features and smoking pack years were characterised using orthogonal distance regression (ODR). We focused on alpha angle features that have previously been demonstrated to correlate with airway obstruction in COPD^6^. Pearson’s product-moment correlation coefficient (*r*) was used to assess the relationships between spirometry metrics and smoking pack years. Comparisons of standardised average breath waveforms were performed using False Discovery Rate (FDR) controlled Mann-Whitney U tests on the pCO_2_ values at each timepoint.

## Results

The analysis cohort comprised 147 participants with COPD stages I–IV, classified according to the 2007 Global Initiative for Chronic Obstructive Lung Disease (GOLD) system. Participants with missing or no previous smoking history or a comorbidity of asthma were excluded from the analysis. The baseline characteristics of these participants are presented in Table 1.

**Table 1:** Demographics and spirometry data of the participants, stratified by smoking status.

	Current smokers (*n* = 21)	Ex-smokers (*n* = 126)	Total (*n* = 147)
Age	62.0 (58.0–68.0)	68.0 (62.0–74.0)	67.0 (61.0–73.0)
Birth sex (female)	13 (61.9%)	56 (44.4%)	69 (46.9%)
BMI (kg/m^2^)	25.3 (23.2–30.1)	25.9 (23.0-32.6)	25.8 (23.0-32.1)
Pack years	36.8 (32.3–45.0)	33.8 (22.5–45.0)	34.0 (22.5–45.0)
Spirometry			
FEV_1_ (L)	2.1 (1.5–2.8)	1.8 (1.3–2.3)	1.8 (1.4–2.3)
FVC (L)	3.2 (2.7–3.7)	3.0 (2.4–3.8)	3.1 (2.4–3.7)
FEV_1_/FVC	0.62 (0.53–0.71)	0.53 (0.36–0.66)	0.56 (0.36–0.67)
Pred. FEV_1_ (%)	62.5 (41.7–77.8)	57.5 (36.0-74.4)	58.0 (37.1–75.7)

Categorical data is represented as a number with its percentage of the total (n (%)). Continuous data is represented as (median (Q1-Q3)).

**Fig. 1:**
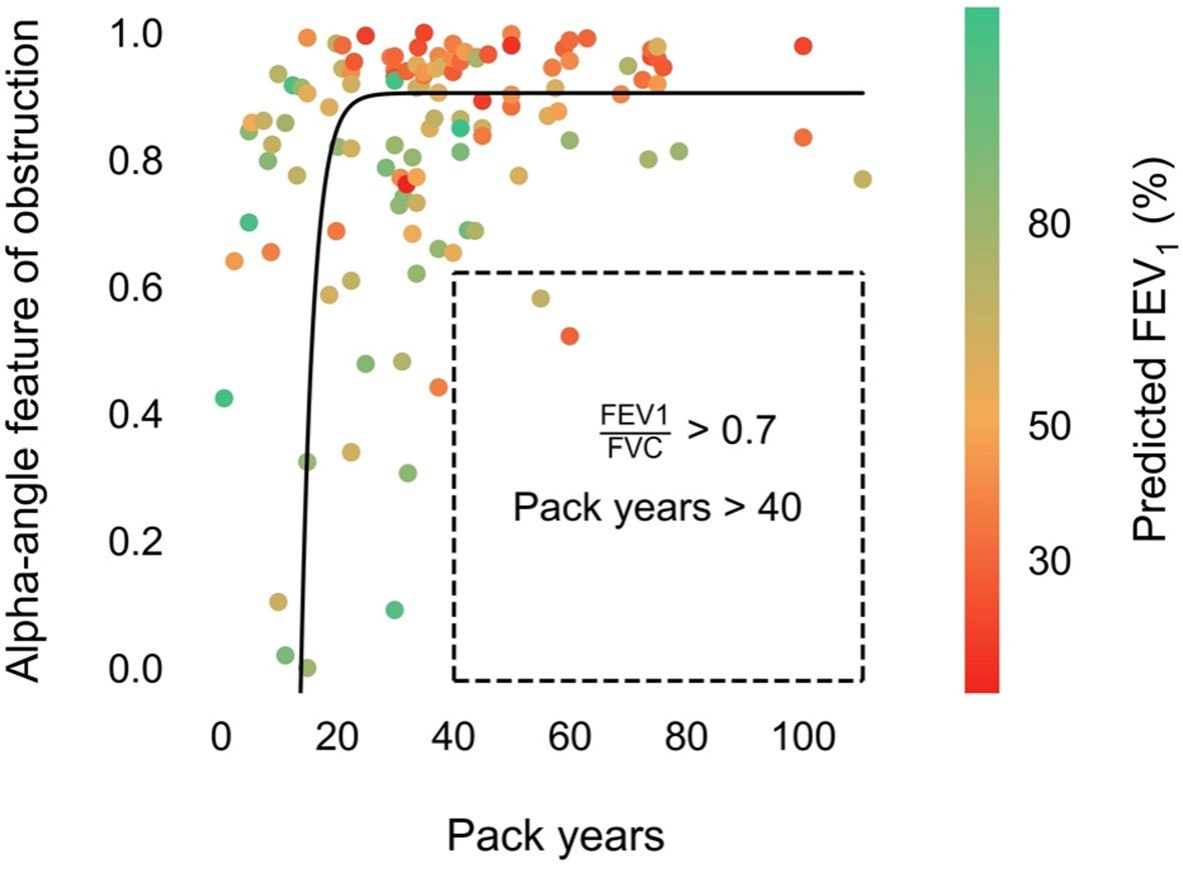
Regression plot of the capnography alpha-angle feature against pack years. Each point represents a patient’s min-max normalised median alpha-angle feature value (lower value indicates better respiratory function) and is coloured by the patient’s corresponding % predicted FEV_1_ value. The dark solid line, obtained via orthogonal distance regression with a single exponential function and grid search, shows the line of best fit. The horizontal line at the top of the boundary box represents the alpha-angle feature value for an FEV_1_/FVC ratio of 0.7, determined by linear regression.

A strong non-linear positive correlation was observed between pack year history and capnography features in the alpha-angle region (Fig. 1). To further investigate this relationship, patients were categorised into two groups: those with fewer than 25 pack years and those with more than 25 pack years; this threshold was chosen as it represents the elbow of the function in Fig. 1. Comparison of the median alpha-angle feature between these groups revealed a significant difference (*p* = 0.011) and a moderate effect size (Cohen’s d = 0.59).

**Fig. 2:**
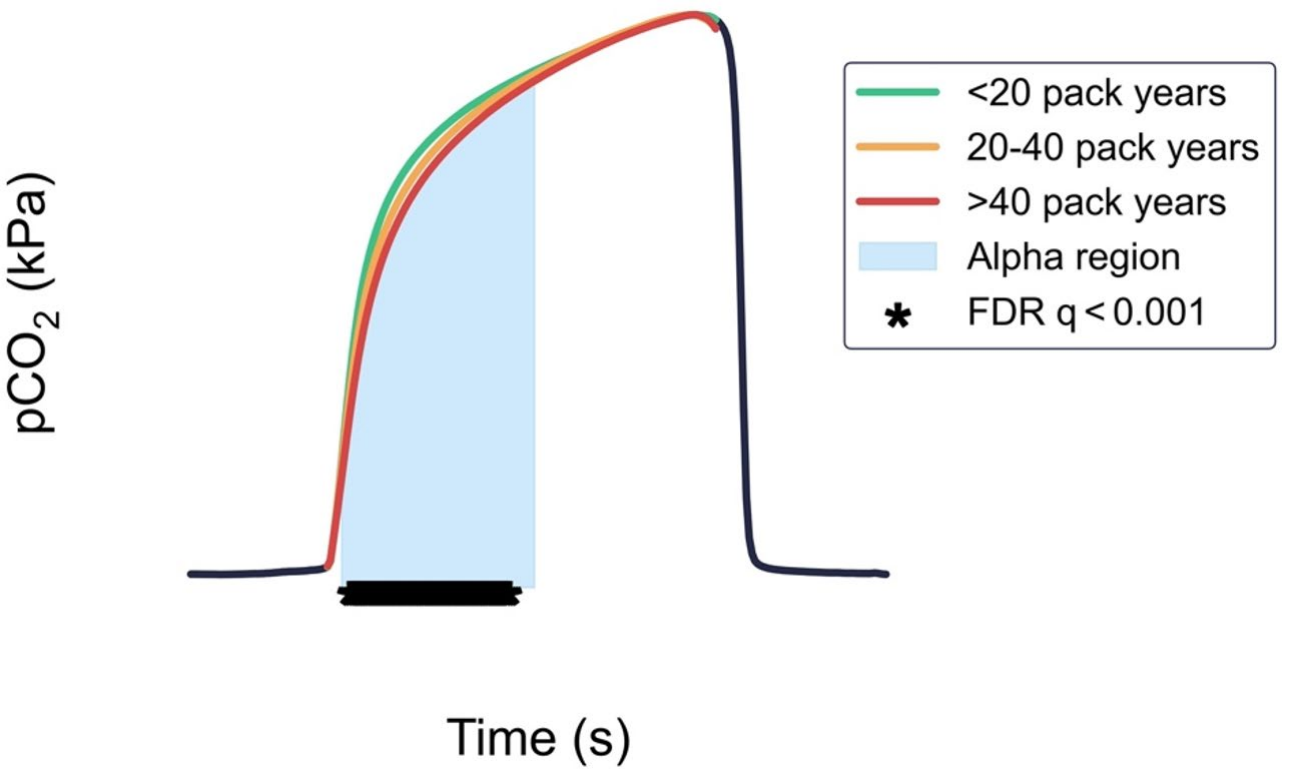
Average breath waveforms of five random patients from each of the < 20, 20–40, and > 40 pack year groups. The highlighted region indicates the alpha angle region of the capnogram. The asterisks indicate datapoints for which the adjusted false discovery rate (FDR) q-values for a Mann-Whitney U test between the pack years < 20 group and the pack years > 40 group were < 0.001.

To visualise the impact of cumulative smoking exposure on capnography waveform shape, ten capnograms were randomly sampled from each patient across the < 20, 20–40, and > 40 pack year groups. Representative waveforms were generated by averaging the capnograms from each group (Fig. 2). These waveforms demonstrate increased alpha region curvature with greater pack year history. Statistical testing showed a significant difference between the pack year < 20 group versus pack years > 40 group (FDR q < 0.001) in the alpha region.

**Fig. 3:**
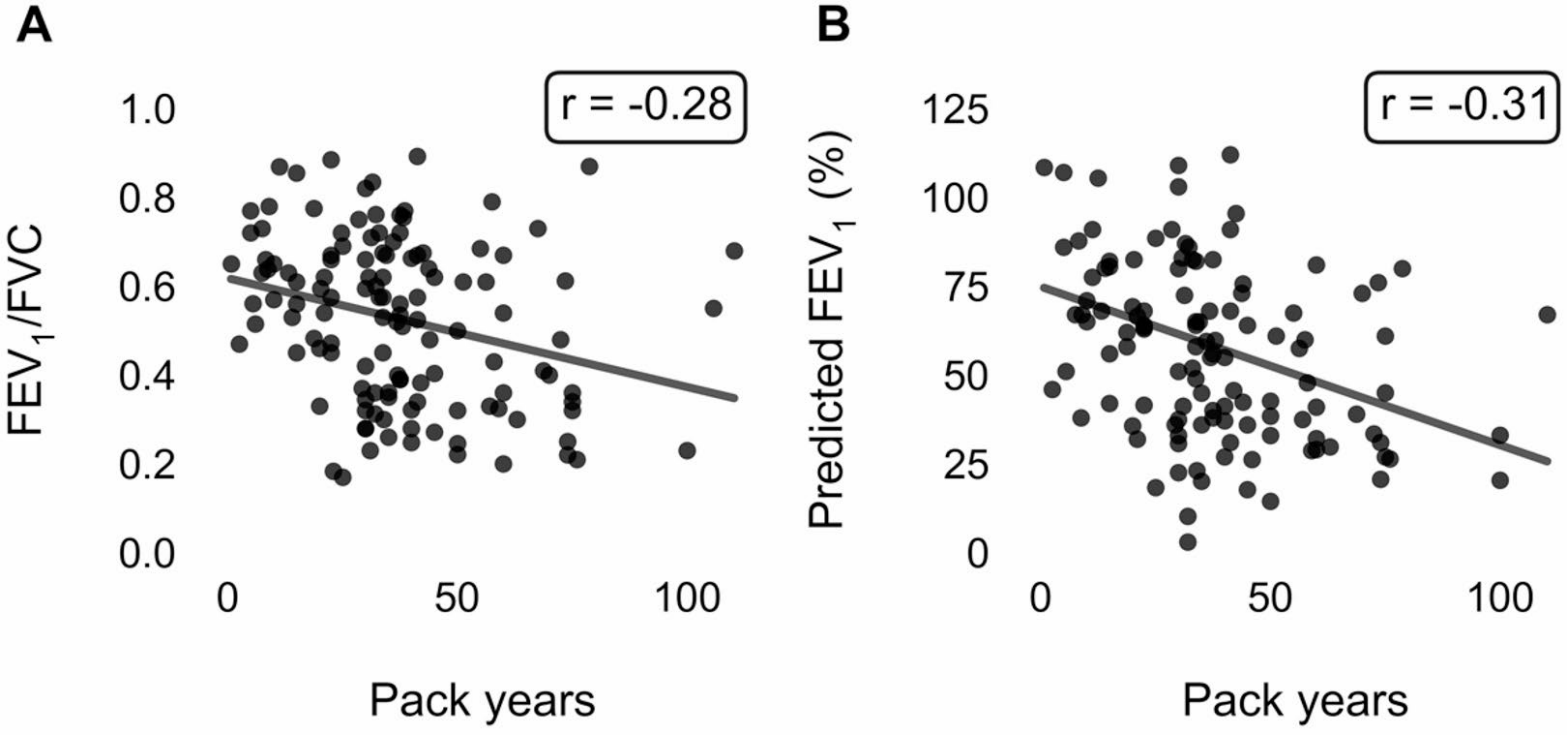
Scatterplots of (**A**) FEV_1_/FVC ratio and (**B**) % predicted FEV_1_ against pack years. Neither plot demonstrates a strong observable trend or inflection point, in contrast to the correlation observed with the capnography feature.

The relationships between pack years and the spirometry measures FEV_1_/FVC and percent predicted FEV_1_ were also assessed, as shown in Fig. 3. There was a weak negative linear association between smoking pack years and both FEV_1_/FVC (*r* = -0.28) and percent predicted FEV_1_ (*r* = -0.31). Importantly, neither variable demonstrated an inflection point at a particular pack year threshold.

## Discussion

The study revealed a strong positive correlation between the capnography alpha angle region and pack years, supporting the hypothesis that airway obstruction and remodelling increases with smoking exposure. Structural airway remodelling in COPD, including epithelial damage and enlargement of submucosal mucous glands, manifests in the capnography waveform geometry, reflecting heterogeneous airflow and uneven alveolar emptying^7^. The alpha angle region of the waveform represents the transition from larger airway gas to alveolar gas and is known to exhibit increased curvature in more severe COPD^6,8^. The alpha angle itself is closely linked to the alveolar (Phase III) plateau region and has been demonstrated to reflect the ventilation/perfusion (V/Q) ratio^9^.

A notable finding was the non-linear association observed that reached an inflection around 25 pack years, potentially indicating a ‘tipping point’ beyond which the likelihood of retaining normal lung function significantly diminishes. Furthermore, all participants with pack year histories beyond 40 pack years had significantly altered CO_2_ waveform geometry. This observation was corroborated by the average capnogram waveforms for the three defined groups with different smoking exposures, which showed greater curvature in the alpha angle region with increasing cumulative smoking exposure. While we also investigated relationships between pack years and spirometry metrics such as FEV_1_/FVC and percent predicted FEV_1_, these did not demonstrate a similar inflection point at a particular pack year threshold. This suggests that capnography may offer unique insights into smoking-related airway changes.

Several limitations should be noted. Primarily, this research only examines smoking-related airway changes in those with a diagnosis of COPD, limiting its generalisability to a general smoking population, as not all people with smoking exposure will develop COPD. Future work will be necessary to understand whether the findings translate to a general smoking population in the context of determining utility for population screening. Additionally, further analysis may be required to understand the relationship between pack years and capnography features in different subgroups, e.g. the newly defined PRISm population^10^. Finally, only features with a strong a-priori hypothesis grounded in known physiologic mechanism were studied; future work could involve developing a multivariate regression model to predict smoking exposure from the whole waveform, not just the alpha angle region.

## Conclusions

The findings demonstrate the ability of the N-Tidal device to detect smoking-related airway remodelling in patients with COPD from CO_2_ waveform geometry, potentially offering a more sensitive measure than traditional spirometry. This capability could, in the future, be extended to estimate a patient’s risk of developing functional airway obstruction to support preventative intervention. Moreover, the delineation of the exposure risk-profile using the N-Tidal capnometer provides valuable mechanistic insights and could potentially be used in the future to inform tobacco cessation strategies.

## Data Availability

The study datasets analysed in the current manuscript are not publicly available due to reasons of patient confidentiality and commercial sensitivity. However, data to interpret, replicate and build upon the findings reported in the manuscript are available subject to confidentiality provisions and consent from the authors’ institutions. Requests for access to the data should be directed to the corresponding author.

## References

[1] Global Burden of Disease Collaborative Network. Global Burden of Disease Study 2019 (GBD 2019) Reference Life Table. Seattle, United States of America: Institute for Health Metrics and Evaluation (IHME). 10.6069/1D4Y-YQ37 (2021).

[2] Goodchild, M., Nargis, N. & Tursan d’Espaignet, E. Global economic cost of smoking-attributable diseases. *Tob. Control*. **27**, 58–64 (2018). 28138063 10.1136/tobaccocontrol-2016-053305 PMC5801657

[3] Jayes, L. et al. SmokeHaz: systematic reviews and meta-analyses of the effects of smoking on respiratory health. *Chest* **150** (1), 164–179 (2016). 27102185 10.1016/j.chest.2016.03.060

[4] Bate, S. R. et al. N-Tidal C: a portable, hand held device for assessing respiratory performance and injury. *Am. J. Respir. Crit Care Med.* **197**, A2371 (2018).

[5] National Institute for Health and Care Excellence (NICE). Chronic obstructive pulmonary disease in over 16s: diagnosis and management. NICE. (2018). https://www.nice.org.uk/guidance/ng115 31211541

[6] Talker, L. et al. Diagnosis and severity assessment of COPD using a novel fast-response capnometer and interpretable machine learning. *COPD: J. Chronic Obstr. Pulmonary Disease*. **21** (1), 2321379 (2024). 10.1080/15412555.2024.2321379 38655897

[7] Raby, K. L. et al. Mechanisms of airway epithelial injury and abnormal repair in asthma and COPD. *Front. Immunol.* **14**, 1201658 (2023). (2023). 10.3389/fimmu.2023.1201658 PMC10374037 37520564

[8] Talker, L. et al. Machine diagnosis of chronic obstructive pulmonary disease using a novel fast-response capnometer. *Respir Res.* **24** (1), 1–11 (2023). 37268935 10.1186/s12931-023-02460-z PMC10239171

[9] Bhavani-Shankar, K. et al. Capnometry and anaesthesia. *Can. J. Anaesth.* **39** (6), 617–632 (1992). 1643689 10.1007/BF03008330

[10] Global Initiative for Chronic Obstructive Lung Disease. 2025 REPORT Global Strategy for the Diagnosis, Management, and Prevention of Chronic Obstructive Pulmonary Disease. https://goldcopd.org/wp-content/uploads/2024/11/GOLD-2025-Report-v1.0-15Nov2024_WMV.pdf (2025).

